# Disseminated Gastrointestinal Basidiobolomycosis (GIB) in an infant from Western India

**DOI:** 10.1016/j.mmcr.2019.10.001

**Published:** 2019-10-03

**Authors:** Anuradha Sharma, Rahul Saxena, Arvind Sinha, Shambhavi Singh, Taruna Yadav

**Affiliations:** a Department of Microbiology, All India Institute of Medical Sciences, Jodhpur, Rajasthan, 342005, India; b Department of Pediatric Surgery, All India Institute of Medical Sciences, Jodhpur, Rajasthan, 342005, India; c Department of Radiodiagnosis, All India Institute of Medical Sciences, Jodhpur, Rajasthan, 342005, India

**Keywords:** Disseminated gastrointestinal basidiobolomycosis, Entomophthoramycosis, GIB

## Abstract

Basidiobolomycosis is an infection due to the fungus *Basidiobolus ranarum*, an environmental saprophyte found worldwide. We are reporting youngest case of Gastrointestinal Basidiobolomycosis (GIB) in a two month old boy from India. He presented with a mass in right iliac fossa with the signs of intestinal obstruction. Histopathology of the lesion showed broad aseptate hyphae. It disseminated to kidneys. Aspirate culture from nephrostomy yielded growth of *B. ranarum*. It is important to differentiate it from mucormycosis to institute right therapy. Culture remains the gold standard for its diagnosis.

## 1 Introduction

Gastrointestinal fungal infections are an uncommon encounter for the surgeons, gastroenterologist, and radiologist alike. The unfamiliarity with this entity, usually leads to much confusion and diagnostic delay [1]. *B. ranarum* is a fungus belonging to the family Entomophthoraceae of the class Zygomycetes and is mainly associated with subcutaneous infections mostly in tropical areas of South America, Africa, and Asia. The first presumed case of Gastrointestinal Basidiobolomycosis (GIB) was reported in 1964 in a six year old Nigerian boy. Subsequently, two cases were reported in 1979 involving the gastrointestinal tract in apparently healthy individuals [2]. Recently, several case reports are published across the world. The diagnostic delay can alter the outcome in these cases.

Here, we report a case of Gastrointestinal Basidiobolomycosis in a two month old male patient who is a resident of Rajasthan, Western India. To the best of our knowledge, this is the first isolation of *B. ranarum* from Western part of India as well as youngest patient ever reported. Its early diagnosis remained challenging due to similarity with other intestinal diseases.

## 2 Case

**Fig. 1:**
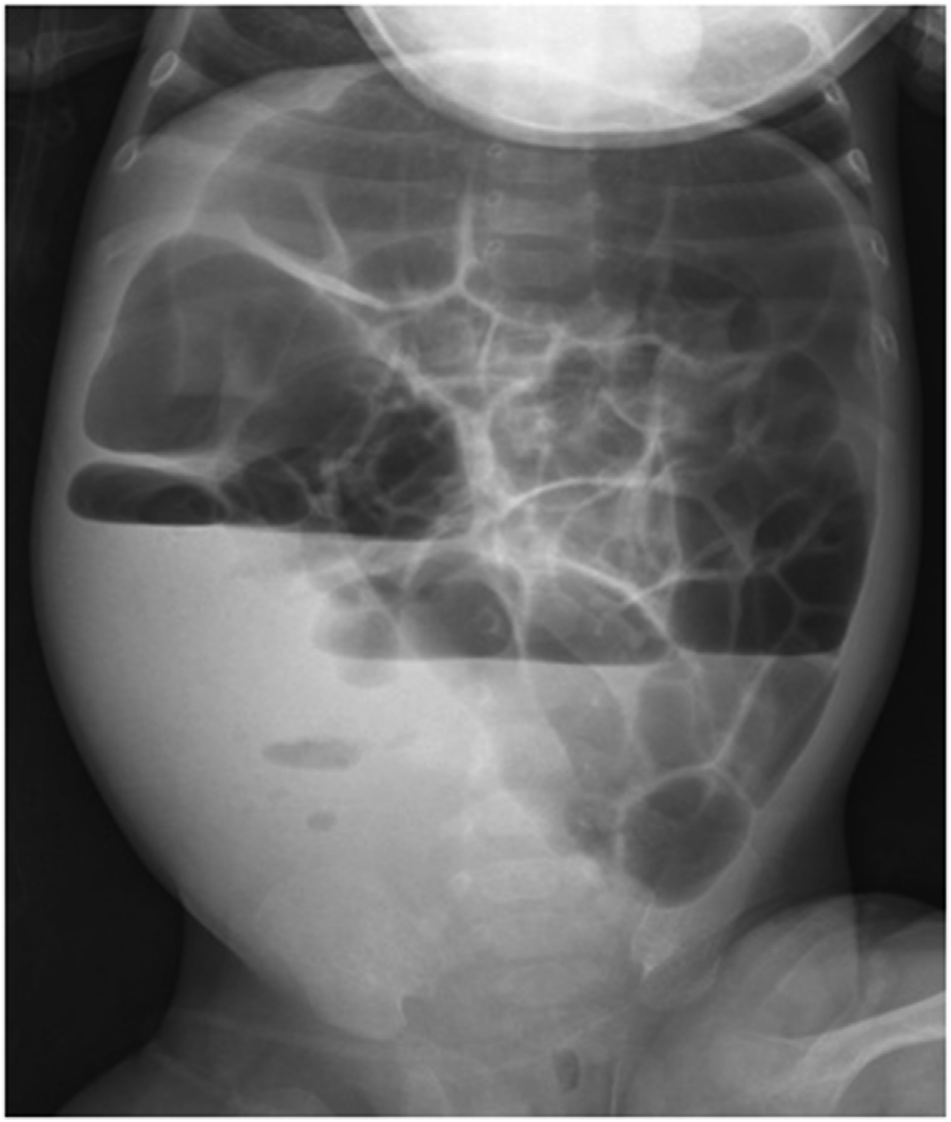
Abdominal radiograph showing features of intestinal obstruction with multiple air fluid levels. A soft tissue density is seen in right iliac and lumbar regions displacing bowel loops to left side. Both small and large bowel loops are dilated.

**Fig. 2:**
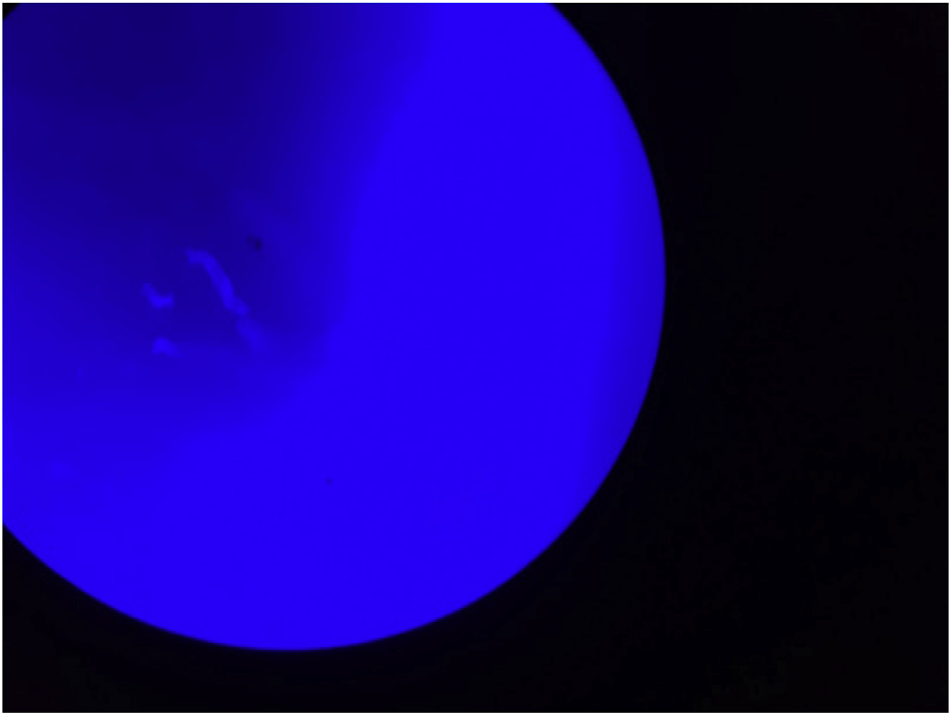
Calcoflour white mount of biopsy tissue (40x).

**Fig. 3:**
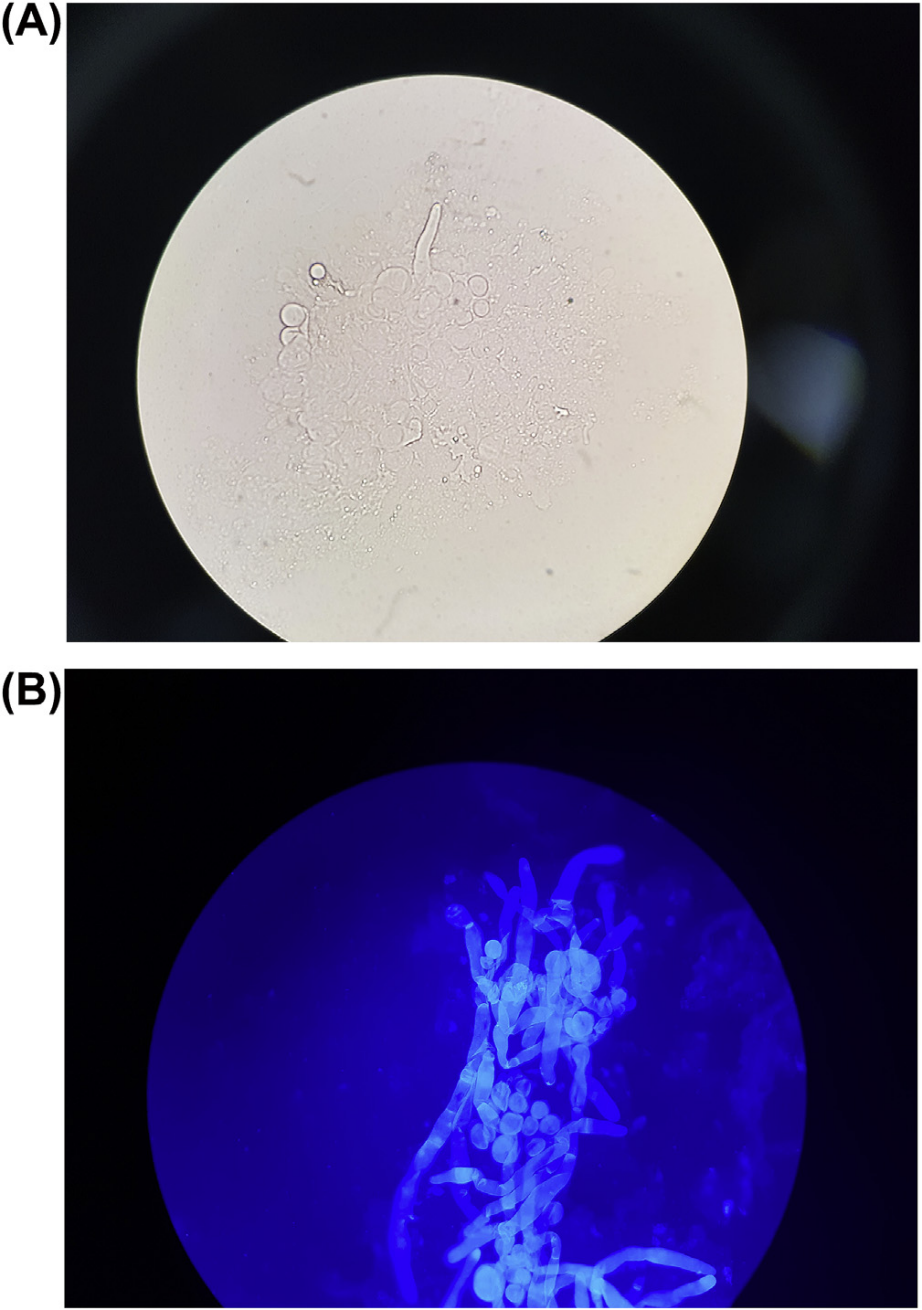
Potassium hydroxide (Fig. 3a) and calcoflour white mount (Fig. 3b) of pus from percutaneous nephrostomy (40x).

**Fig. 4:**
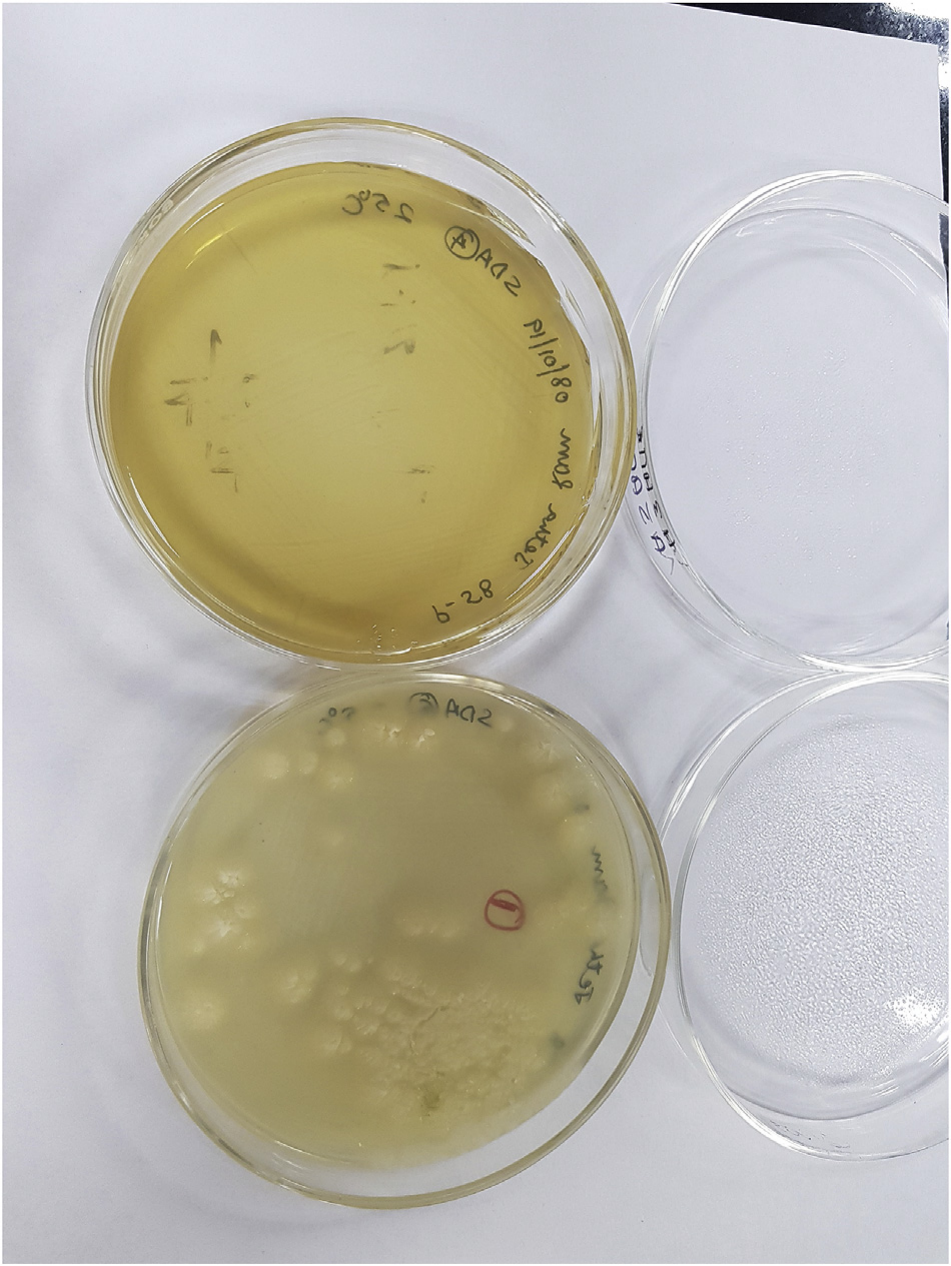
Colony morphology on Sabouraud's dextrose agar (SDA), there is no growth on SDA with actidione.

A two month old male child from the deserts of western part of Rajasthan, India presented to our OPD with the complaint of abdominal swelling associated with vomiting (day+0). Patient had one day history of high grade fever with decreased urine output and decreased activity. There was a history of three day hospitalization at the time of birth for poor feeding. It was a normal, full term delivery in the hospital. On abdominal examination, a mass was palpable, firm in consistency, free from underlying structures, along the right side of abdomen from right iliac fossa (RIF) to just two fingers below the ribs, medially extending towards the stomach. Radiography (day+1) and Contrast Enhanced Computed Tomography of abdomen (day+5) showed large heterogeneously enhancing abdomino-pelvic mass predominantly in pre-sacral and right inguinal fossa (RIF) region with encasement of sigmoid colon and upper part of rectum, with resultant bowel obstruction (Fig. 1). It was reported as an aggressive malignant mass. Other significant investigations showed hsCRP – 94.41 mg/L; WBC count – 19.86 × 10^3^/μL with neutrophils (41%), lymphocytes (30%), monocytes (8.5%), and eosinophils (10.5%). Intraoperative biopsy (day+23) was collected which was suggestive of chronic necrotizing granulomatous inflammation. No underlying evidence of malignancy was seen in the sections examined. A second exploratory biopsy (day+53) at another hospital showed scanty broad fungal profiles and foreign body giant cell reaction. In our microbiology laboratory, a biopsy tissue (day+73) was sent for KOH mount which showed broad, hyaline, aseptate hyphae (Fig. 2). Culture did not yield any growth as the sample was sent in formalin. Microscopy was suggestive of mucormycosis and patient was treated with amphotericin B. Despite treatment patient developed pyonephrosis within a month. Abscess drained from percutaneous nephrostomy was sent to microbiology laboratory (day+97). On microscopic examination broad, hyaline, aseptate hyphae with wide angle branching and papillated conidia were seen (Fig. 3a and b). On Sabouraud's dextrose agar, within 24–48 hours, it showed wrinkled, yellowish-white, waxy, furrowed colonies with radial folds (Fig. 4). Lactophenol cotton blue mount of culture showed broad hyaline aseptate hyphae with conidia of various sizes, globose to pyriform, smooth walled having granules inside, suggestive of *B. ranarum.* The culture was further confirmed at National Culture Collection of Pathogenic Fungi (NCCPF), Postgraduate Institute of Medical Education and Research (PGIMER), Chandigarh, India by sequencing (*Basidiobolus ranarum* 18S ribosomal RNA gene, partial sequence; internal transcribed spacer 1, 5.8S ribosomal RNA gene and internal transcribed spacer 2, complete sequence; and 26S ribosomal RNA gene, partial sequence). (ACCESSION AY211271).

**Fig. 5:**
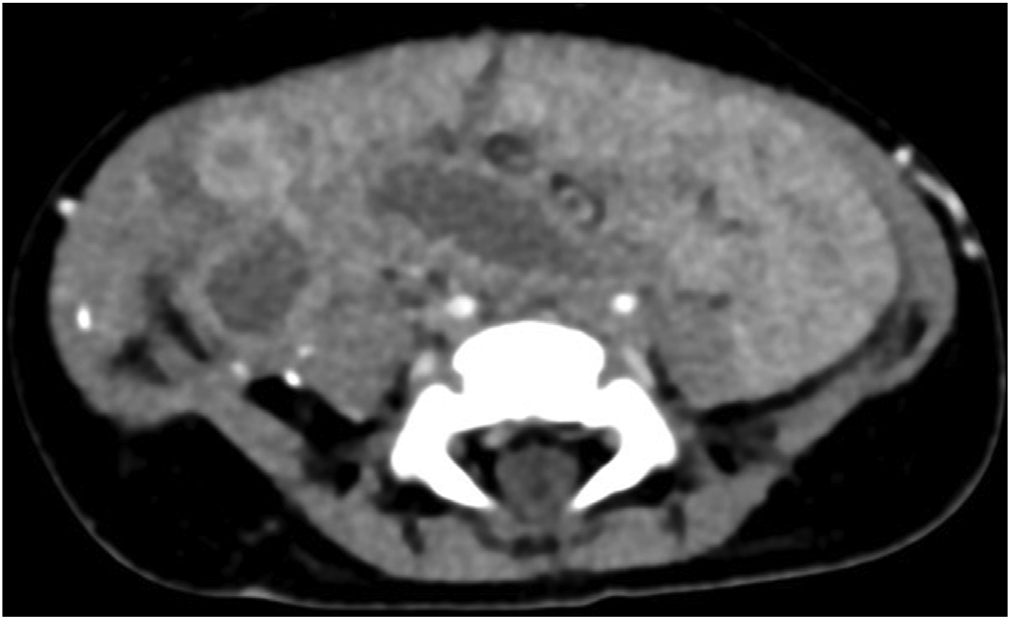
Follow up scan after six months shows decrease in the size and extent of right inguinal fossa (RIF) mass lesion.

Isolation of entomophthorales led to change in the treatment, amphotericin was stopped and itraconazole (25 mg twice a day, per orally) was started. Therapeutic drug monitoring was performed after two weeks on trough sample, it did not reveal anything as sample was inadequate. Ileostomy and nephrostomy were performed to manage patient clinically. Blood and urine cultures remained sterile. Mass was not removed surgically because of extensive entrapment of intestines. Patient could not be given saturated salt solution of potassium iodide because of unexplained continuous hyperkalemia (6.69 mmol/L), otherwise it is a cheap and rapidly effective formulation for this entity. After six months of treatment with oral itraconazole, radiologically there was significant reduction in the size of the mass (Fig. 5) and eosinophil count had also become normal.

## 3 Discussion

Infections caused by Entomophthorales include both conidiobolomycosis and basidiobolomycosis with the latter being more common. It is presumed that infection is acquired after minor trauma to skin or after insect bites. The Entomophthorales are true pathogens, infecting primarily immunocompetent hosts. Most patients are in apparent good health before acquiring infection. Basidiobolomycosis is a disease caused by the fungus *Basidiobolus ranarum*, an environmental saprophyte found worldwide [3,4]. It has been reported from South India involving extremities, trunk, intestinal tract and rarely other parts of the body. The disease usually occurs in children, less often in adolescents and rarely in adults [5]. It seems that Entomophthoramycosis has correlation to age. Conidiobolomycosis is uncommon in children, but 88% of basidiobolomycosis cases occur in patients younger than 20 years of age [6]. Patients with *B. ranarum* infection may present with subcutaneous, gastrointestinal, or systemic lesions. Recently, its etiologic role in gastrointestinal infections has been increasingly recognized. Symptoms include fever, abdominal pain, diarrhea, constipation, weight loss, and rarely, chills and rigors. Gastrointestinal Basidiobolomycosis (GIB) poses diagnostic difficulties, as its clinical presentation is nonspecific, with no identifiable risk factors [7].

Subcutaneous infection manifests clinically as a firm, painless, disciform nodule on the trunk, buttocks or extremities (“bathing suit distribution”), which if untreated may enlarge and spread locally, but systemic dissemination is extremely uncommon [1]. It is proposed that it affects male children more commonly because of using plant material to clean themselves after defecating [8]. In a study of ten cases of Entomophthoramycosis by Krishnan et al., eight were caused by *Basidiobolus haptosporus* (now known as *B. ranarum*) predominantly in children below ten years of age [9].

Entomophthoramycosis is a potentially curable disease which can masquerade as a neoplasm. *B. ranarum* organisms generally do not invade blood vessels and rarely disseminate. However, van den Berk et al. have presented a case of obstructing colon tumor with associated liver mass and Bigliazzi et al. have presented a case of disseminated basidiobolomycosis in an immunocompetent woman, with lung involvement as the first clinical manifestation. The prognosis of GIB is usually favorable; however, both these cases had fatal outcomes [4,10]. In a similar case report by Sriram Krishnamurthy et al., a seven year old boy presented with a chronic, indurated, tender left thigh swelling in association with a hypertensive emergency. He had a bilateral moderate degree of hydronephrosis and a left perinephric abscess, and Magnetic Resonance Imaging features of posterior reversible encephalopathy syndrome. Histopathological examination of the biopsy specimen demonstrated eosinophilic fasciitis with filamentous fungi. *Basidiobolus ranarum* was isolated from the culture. The fungus was also isolated from perinephric fluid aspirate [11].

Literature has more than 71 cases reported of GIB with age range 1.5–80 years, four cases with recurrence and eight deaths. It involves stomach, small intestine and colon. It can disseminate to liver, pancreas and renal system. It may present with complications like bowel perforation, obstructive uropathy, esophageal varices, duodeno-biliary fistula or even death. Marked eosinophilia is seen in these cases [12,13]. Endoscopic biopsies are not helpful as fungus lies deep beneath the mucosa. Culture remains the gold standard to diagnose entomophthoramycosis. Histology reveals Splendore-Hoeppli phenomenon. DNA probes and PCR are being more commonly used in affluent countries. While amphotericin B remains cornerstone therapy for mucormycosis, it has negligible role in the treatment of entomophthoramycosis [13]. Surgery plays a selective role depending on the site of infection as there can be recurrence locally [8].

## 4 Summary

Gastrointestinal basidiobolomycosis is often misdiagnosed as cancer, tuberculosis or inflammatory bowel disease. Demonstration of broad, pauci-septate fungal hyphae from the tissue and confirmation by culture clinches the diagnosis. Treatment with potassium iodide and azoles gives good clinical outcome. Our experience with this case highlights the importance of awareness and early recognition of this condition to prevent further advancement of disease, misdiagnosis, and avoidable surgical interventions.

## Declaration of competing interestCOI

None.
